# Revealing porphyry mineralization Cu-Au signatures via analyzing aeromagnetic and remote sensing data of Dara-Monqul, area Egypt

**DOI:** 10.1038/s41598-025-09809-y

**Published:** 2025-07-24

**Authors:** Mahmoud Abdellatif, Sayed O. Elkhateeb, Mahmoud Abd El-Rahman Hegab, Ali Shebl, Ghada Mohamed, Ali M. Mahdi

**Affiliations:** 1 Geology department, Faculty of Science, South Valley University, Qena, Egypt; 2 Authority for Remote Sensing and Space Sciences (NARSS), Cairo, Egypt; 3 Geology department, Faculty of Science, Tanta University, Tanta, Egypt; 4 Mineralogy and Geology Department, Debrecen University, Debrecen, 4032 Hungary

**Keywords:** Aeromagnetic, Remote sensing, Porphyry Cu-Au, Potential mineralization, Geology, Geophysics

## Abstract

In this paper, an efficient strategy based-first link between aeromagnetic and remote sensing data is presented to delineate the signatures associated with mineralization, especially the porphyry-style one in Dara-Monqul area, Northeastern Desert, Egypt. Initially, aeromagnetic derivative filters like analytical signal (AS), first vertical derivative (FVD) and Euler deconvolution revealed the NW-SE as a preferred direction for mineral occurrence, with other traces of NE-SW, NNE-SSW and N-S trends. The depths of these trends range from 0 to 0.8 km. Exploration targeting (CET) grid and porphyry analysis had an operative role in mapping the structure complexity, Dykes and Porphyry features. Lithological discrimination and hydrothermal alteration (ferrous silicates, hydroxyl, phyllic and potassic) have been mapped by applying various image processing techniques of Remote sensing data, including False Color Composite (FCC), Principal Component Analysis (PCA), Independent Component Analysis (ICA), Minimum Noise Fraction (MNF), and band ratio (BR). Regarding these results, two distinct zones occupied Monqul and Dara regions have been indicated with high potentiality mineralization (Cu-Au), with constructing a composite potential mineralization (CPM) map. Field observation and Scanning Electron Microscopy (SEM) have been implemented to verify the exact locations and analyze the mineral chemistry of gangue and ore minerals within the two promised zones.

## Introduction

Hydrothermal mineral deposits are generated as a result of hydrothermal fluid activities and their interaction with the surrounding rocks. Changes in temperature and pressure conditions made these solutions possible to react with wall rocks, so the contained minerals from hot solutions concentrated to compose these types of deposits. They are localized inside cracks, fissures, and other accessible apertures in host rocks^1,3^. Based on the mode of occurrence and the depositional environment, the hydrothermal deposits are usually categorized into porphyry, vein, skarn, volcanogenic massive sulfide, sedimentary exhalative, and Mississippi-Valley deposits^1^.

With regard to porphyry-style mineralization, it is considered the most valuable and has the highest priority in exploration, since they are the world’s main source of molybdenum(Mo), an essential source of copper (Cu), a major source of gold (Au), silver (Ag), and other byproduct metals^4,5^. Consequently, they are in great demand. Additionally, the most prevalent forms of hydrothermal alteration linked to porphyry intrusions are Propylitic (chlorite), argillic (clays), phyllic (sericite), and potassic (k-feldspar)^6,7^. The occurrences of porphyry types are commonly concentrated in North and South America, Southeast Asia, southern central Europe, eastern Turkey, some areas in China, Namibia and Zambia in Africa^8^. In the Arabian Nubian Shield (NE Africa), they have not been appropriately explored However, the potentiality of these deposits is highlighted in Sudan (Jebel Ohier porphyry Cu deposit)^9^. Egypt is distinguished by its significant mineralization potential, particularly for Orogenic, Banded Iron Formation (BIF), and Volcanic Metasediment (VMS) gold in the Eastern Desert^10^. Some studies in a number of regions have made it possible to identify the type of porphyry mineralization (Cu ± Au ± Mo), such as El Samra region situated Southeast of Sinai^11^, Wadi Ranga^12^, Hamash^13^ located in the southeastern desert and Um Balad, Wadi Dara, and Gabal Monqul regions in the northeastern desert^14,17^.

There has always been a great interest in research and prospecting for economic raw materials, especially porphyry deposits, with tremendous progress this has become easier and more widespread. Several types of data (e.g., geological, geochemical, Remote sensing, and airborne geophysical) with different approaches and enhancement techniques can be utilized for this target^12,15,17^. Remote sensing is widely used for lithological mapping, hydrothermal alteration detection, and mineral potential mapping. Its strength lies in identifying alteration zones through minerals with distinct spectral signatures, especially in the VNIR and SWIR regions. For example, muscovite linked to phyllic alteration, shows characteristic absorption features near 2200 and 2350 nm in the SWIR range^18,20^. However, detecting such minerals does not confirm mineralization, as they may also form through weathering or metamorphism^20^. Thus, geological validation is essential, though clusters of muscovite, epidote, and kaolinite often suggest mineral potential. Several integrated remote sensing datasets, including ASTER, Sentinel-2, and PRISMA hyperspectral are usually applied to better characterize the rock units and highlight these potential alteration zones. These datasets were chosen for their proven effectiveness in various geological applications, including lithological mapping^21,22^, hydrothermal alteration detection^20,23,25^, textural analysis^26^, and structural mapping^27,28^, delineation base-metal^22,29^. As a diagnostic tool for the early phases of porphyry-type deposits, multispectral and hyperspectral remote sensing data can identify hydrothermal alteration minerals with diagnostic spectral absorption features in the visible and near-infrared through the shortwave length infrared regions^4,17,30−38^. Also, aeromagnetic surveys provide important information for comprehending the subsurface structure and identifying prospective mineralization zones, helping to define geological structures^20,24^. Porphyry-style mineral deposits provide excellent examples of magnetic properties linked with alteration, as broad regions of hydrothermal alteration frequently exhibit different magnetism than parts of unaltered geology^39^.

For our investigated area, several studies recently have been introduced regarding the occurrences of porphyry mineralization (South Gabal Monqul) through analysis of geological, Geochemical, and remote sensing data^12,33,40,41^. On the basis of the previous results and the first linkup between aeromagnetic, variety of remote sensing data (including ASTER, Sentinel-2, and PRISMA hyperspectral), field observation, and Scanning Electron Microscopy (SEM) analysis, we could provide a significant view about the potential porphyry mineralization signatures not only of Gebel Munqle but also the surrounding areas. This view can be successfully accomplished by providing texture, phase, lineation, alteration, field observation, and SEM analysis.

## Geological setting

**Fig. 1:**
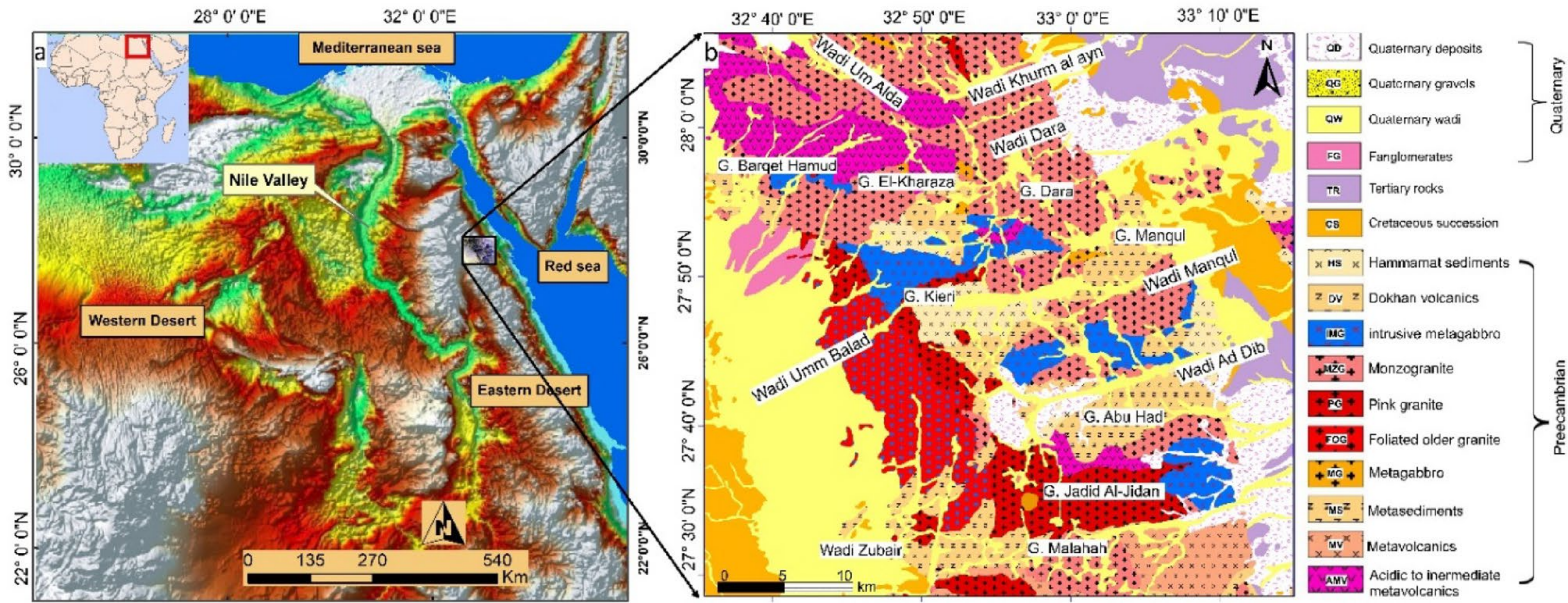
(**a**) Location map of the study area, (**b**) Geological map of the Dara - Monqul area, Northern Egyptian Eastern Desert modified after^45^. (Created by SmartSketch v. 4.0 software; https://smartsketch.software.informer.com/4.0/*).*

The research area is bordered by latitudes 27^o^ 30′ and 28^o^ 02′ N and longitudes 32^o^ 36′ to 33^o^ 14′ E in the northern part of the Egyptian Eastern Desert in the Ras Gharib segment (Fig. 1a). The Ras Gharib segment is divided into two rock suites: (1) the island arc suite (770 − 719 Ma) with metavolcanics, diorite, trondhjemites, and granodiorite, and (2) the post-collisional suite (630 − 590 Ma) with Dokhan volcanics, pyroclastics, Hammamat sediments, monzogranite to syenogranite, and alkali feldspar granite^42,43^.

The geology of the area is predominantly characterized by older and younger granitic rocks, Dokhan volcanics, and Hammamat sediments, as determined through the geological map (Fig. 1b) and field observations^16^. To characterize the geological and structural setting of the study area, multiple datasets were employed. A high-resolution geological map (scale 1:100,000) was used to delineate lithological units and major structures. Exposures of older country rocks, such as serpentinites, metavolcanics, and metagabbro-diorite, are relatively rare and mainly located in the northern and southern parts of the study area. Younger granitoids are intruded into the pre-existing rocks, while older granitic rocks are extruded by the Dokhan volcanic. The metavolcanic are thrust over serpentinites and intruded by both monzogranites and metagabbro-diorites through distinct intrusive contacts. Dense sequences of conglomerate, sandstone, graywacke, and siltstone belonging to the Hammamat sediments unconformably accompany the Dokhan volcanic^40^.

At Wadi Monqul, the contact between the Dokhan volcanics and Hammamat sediments is marked by prominent fault strikes trending 90° and 60° south. The granodiorite and subvolcanic Dokhan volcanics cut through a volcano-plutonic association of arc metavolcanic and volcaniclastic (metasedimentary) rocks. The Hammamat sediments, distinguished by thick sequences, are unconformably connected with the Dokhan volcanics and are widely dispersed across the research area^41^. Hornblende gabbro and porphyritic biotite granite intrude into both the Hammamat sediments and surrounding rocks. The older granitoid rocks, mainly granodiorites, occur in low-lying areas and are characterized by moderate shearing and foliation along fault planes and shear zones, dipping 35°E and trending NNW^41^. The younger granites are composed mainly of monzogranite to syenogranite and alkali feldspar granite^17,43^. Quaternary sediments, including fanglomerates, wadi deposits, and gravels, dominate the southwestern and northeastern parts of the study area.

The study area is notably prospective for mineralization, primarily associated with hydrothermal processes linked to intrusive and volcanic activities. Gold mineralization is often found along shear zones, faults, and contact between granitic intrusions and metavolcanic sequences^17,41^. Additionally, occurrences of base metals, such as copper and lead, have been reported in association with altered metavolcanic rocks and Dokhan volcanics. Quartz veins and veinlets, often enriched with sulfide minerals, are common along the major fault systems.

Lithology and structural elements play a valuable role in controlling mineralization within the research area. The competency contrast between the intrusive granites and surrounding metavolcanics, combined with the development of shearing zones, faults, and fracture networks, provided pathways for mineral-bearing fluids^42,44^. The faults trending NNW–SSE and ENE–WSW act as major conduits for mineralizing fluids, resulting in the concentration of mineralization along these structural corridors. Hydrothermal alteration zones, mainly silicification, sericitization, and chloritization, are closely associated with mineralized structures^41^.

## Datasets

### Aeromagnetic data

**Fig. 2:**
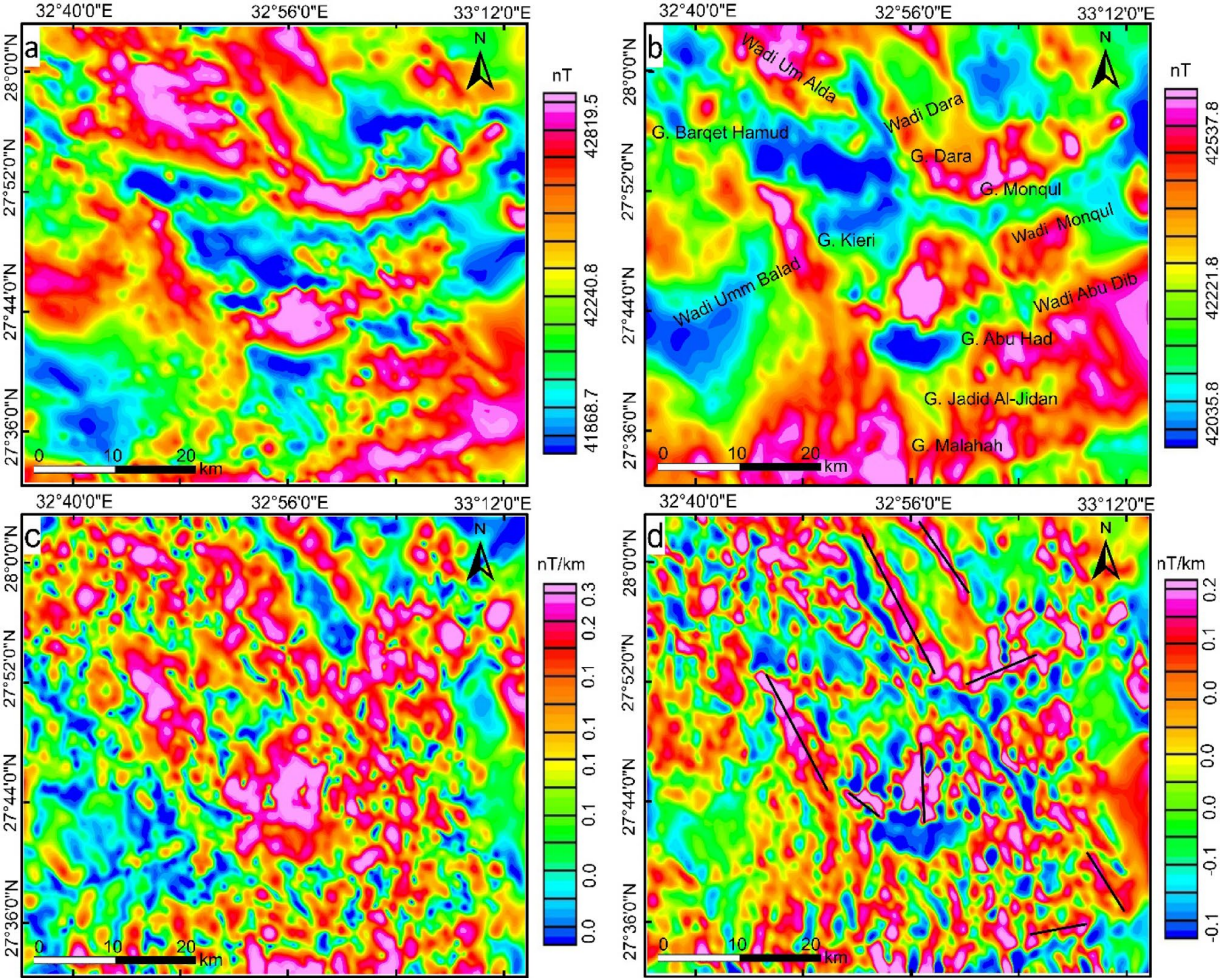
Aeromagnetic data (**a**) Total magnetic intensity map (**b**) Reduced to magnetic pole (RTP) map continued upward by 200 m, (**c**) Analytic signal map, (**d**) First vertical derivative map. Created by Geosoft Oasis Montaj 2015 v. 8.3.3 software; https://www.seequent.com/help-support/oasis-montaj/*).*

The Total magnetic intensity (TMI) map of the study area (Fig. 2a) represents a part of the aeromagnetic survey project conducted by the aero-service division of Western Geophysical Company of America^46^. The survey covered a large section of the north and central Red Sea Mountains at Eastern desert of Egypt. This project is an evaluation program for mineral, groundwater, and petroleum exploration. The flight specifications that were used in the survey are as follows: (i) Flight altitude is 120 m terrain clearance; (ii) Flight line direction is NE-SW, (iii) Traverse flight line spacing is 1.5 km, (iv) Tie line spacing is 10 km, and (v) Twin engine Cessna-Titan, type 404 aircraft. The TMI data is reduced to the magnetic pole (Fig. 2b) to place the magnetic anomalies directly over their causative sources for further processing.

### Remote sensing data

To capture solar radiation across 14 spectral bands, the ASTER sensor was deployed in 1999. ASTER records reflected radiation in six bands ranging from 1.6 to 2.43 μm, with a spatial resolution of 30 m in the shortwave infrared (SWIR) region, and in three bands between 0.52 and 0.86 μm in the visible and near-infrared range with a 15-meter resolution. Additionally, it detects emitted radiation in five bands spanning from 8.125 to 11.65 μm (thermal infrared, TIR region) with a resolution of 90 m.

For better spatial details (up to 10 m) which are recommended for similar geological applications, Sentinel-2 imagery, from the satellite mission (S2A) launched in 2015, was employed in this study. Thirteen spectral bands make up the Sentinel 2 A MSI data. Three bands of aerosol- B1, water vapor- B9, and cirrus - B10 are excluded from the current research. Four red-edge bands including B5, B6, B7, and B8a, two SWIR (B11 and B12) bands, and four (B2, B3, B4, and B8) VNIR bands were utilized.

To enhance the spectral characteristics, PRISMA hyperspectral data were applied. The Italian Space Agency (ISA) launched the PRISMA satellite in March 2019. This satellite uses a prism-based push broom scanning method to record 239 hyperspectral bands, encompassing 66 VNIR bands, 173 SWIR bands, and nine overlapping bands, ranging from VNIR to SWIR. The average spectral resolution is 13 nm and 11 nm (VNIR and SWIR respectively) and it varies ± 2 nm across track^47^ and covers an area of 30 km by 30 km with a 30 m pixel size. All ASTER, Sentinel-2, and PRISMA scenes were selected with 0% cloud cover to ensure optimal data quality for analysis. All satellite datasets underwent standard preprocessing to ensure consistency and accuracy in analysis. Sentinel-2 (S2A MSI) data was acquired cloud-free from ESA, georeferenced to UTM Zone 36 N (WGS-84), and atmospherically corrected using the Sen2Cor processor in SNAP. ASTER (AST_L1A) data was geometrically corrected and atmospherically corrected using the FLAASH module in ENVI, then clipped to the study area. PRISMA Level 2D data, which provides surface reflectance, was also geometrically corrected and subset to the study extent. These steps ensured that all datasets were radiometrically and geometrically aligned for effective lithological, structural, and alteration mapping.

The selection of ASTER, Sentinel-2, and PRISMA datasets over other sensors, such as Landsat-8, was based on their superior spectral and spatial capabilities, particularly in the context of geological and alteration mapping. In a recent comparative study^20^, the performance of these sensors was evaluated, and results indicated that ASTER and Sentinel-2 outperformed Landsat-8 in delineating hydrothermal alteration zones. ASTER is well established in geological remote sensing due to its comprehensive SWIR band suite, which enables the effective identification of alteration minerals, especially in arid and semi-arid terrains. Sentinel-2, with its high spatial resolution (up to 10 m) and wide spectral coverage, provides enhanced discrimination of lithological boundaries and surface alteration features. Additionally, PRISMA, as a hyperspectral sensor, offers detailed spectral information across numerous narrow bands, facilitating more accurate separation of lithological units and improving the recognition of subtle structural features. Collectively, these datasets played a crucial role in resolving the structural framework, lithological heterogeneity, and hydrothermal alteration patterns of the study area.

## Enhancement techniques

### Aeromagnetic data

#### Upward continuation, gradients and Euler techniques

The upward continuation is a form of potential field filter that allows for the observation of deep magnetic sources by amplifying long-wavelength characteristics and attenuating short-wavelength (shallow) ones. The filter enables the recalculation of the magnetic field estimated on one observation plane at a particular height on other higher elevations^48^. At higher elevations (h), the upward continuing magnetic anomaly (∆F) is given as

$$\:\varDelta\:F=\frac{h}{2\pi\:}\iint\:\frac{\varDelta\:F\:\left(x,y,z=0\right)dxdy}{{\left(\right(x-{x}_{0})}^{2}-{\left(y-{y}_{0}\right)}^{2}+{h}^{2}{\left)\right)}^{3/2}}$$

(x0, y0, z0 = -h); x, y, z components at the upward continued level; (x, y, z = 0); x, y, z component at the observed field (mean-sea level).

Derivative techniques in terms of total gradients “analytic signal”^49,50^, and first vertical derivate have been also used on this study to distinguish the edges of magnetic anomalies and delineate the shallow structures respectively. Also, the Euler deconvolution method^51^ is utilized to estimate the depths of area structures (e.g., contact).

#### Structural complexity detection

^5^Proposed a recent Centre for exploration targeting (CET) grid analysis technique for mineral exploration. The technique is considered a based image enhancement approach that involves a group of filters for texture, phase and lineation analysis. Texture analysis is utilized for evaluating faint magnetic response, enhancing local intensity contrasts. Structure detection is valuable in recognizing linear discontinuous, corresponding with lithological boundaries, structures like faults, dykes for understanding geological setting of the area and criteria controls mineralization.

#### Porphyry detection

Porphyry detection is another image analysis approach developed by^52^ for detecting porphyry mineralized systems (e.g., Cu and Au). Hydrothermally altered signatures well related to porphyry-mineralized patterns usually embrace circular or near- circular alterations zones surround a circular like intrusion. The mapped alteration zones generally exhibit an inner zone that is characterized by positive or high magnetic anomalies while the outer one shows a low magnetic response. The method utilizes a circular feature detection technique to emphasize circular to semi-circular anomalies. The borders of the circular features are detected by Amplitude contrast transform filter, and then a boundary tracing tool outlines the detected porphyry-like features.

### Remote sensing

**Fig. 3:**
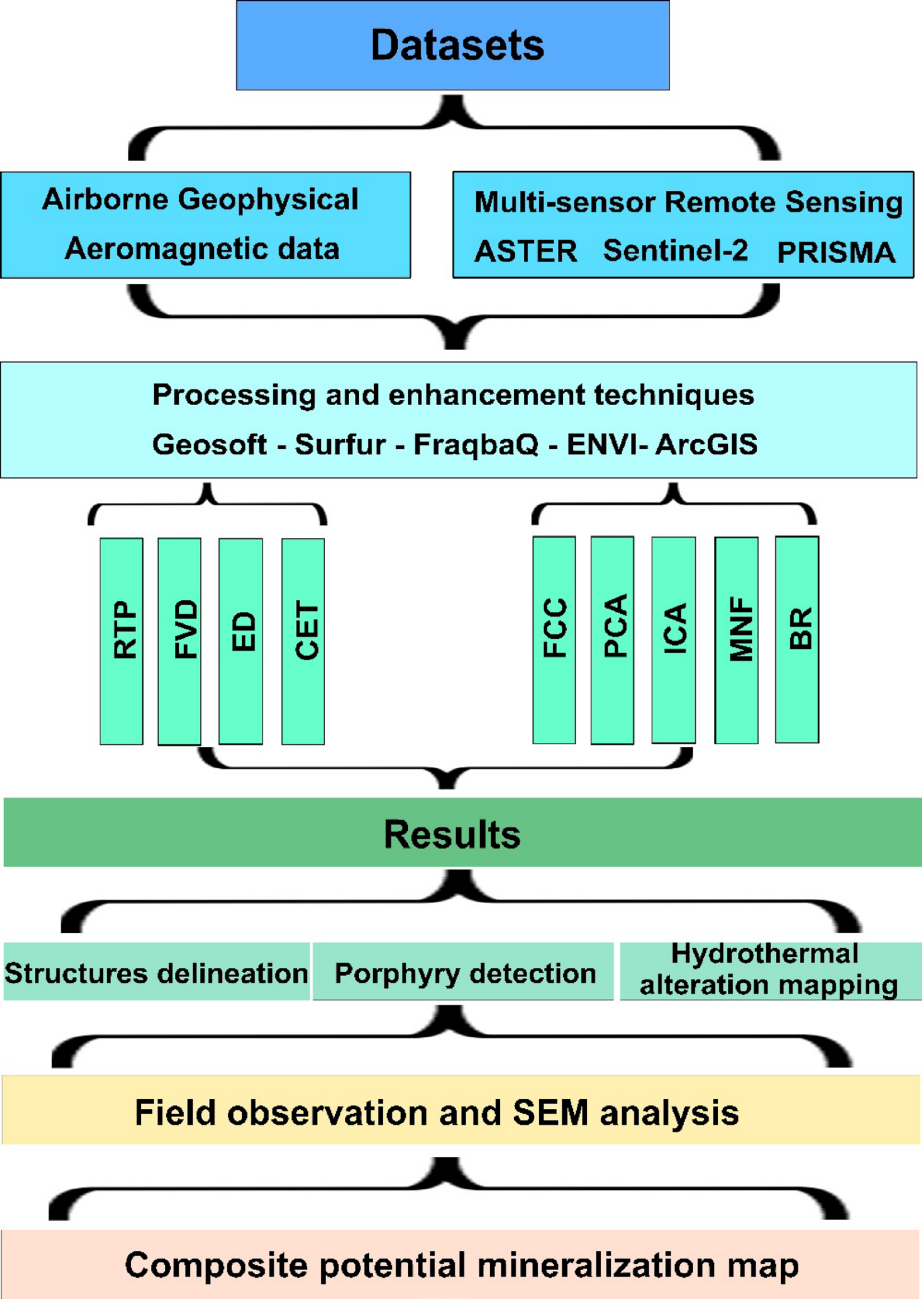
Flow chart approach used in the current study.

Various image processing techniques including False Color Composite (FCC), Principal Component Analysis (PCA), Independent Component Analysis (ICA), Minimum Noise Fraction (MNF), and Band Ratio (BR) were applied to these different datasets. The aim was to enhance the lithological separation between the exposed rock units and highlight the main hydrothermal alteration zones employing these various spectral and spatial characteristics. Despite its simplicity, FCC remains one of the most widely used techniques in geological studies, as it enables effective differentiation of exposed rock units. The amount of information obtained depends on the selected bands (spectral properties) and the target rocks under investigation. In general, for geological applications, it is recommended to include at least one SWIR band^53^. Principal Component Analysis (PCA) serves as a powerful tool for simplifying the complex nature of remote sensing data, particularly when it comes to mapping different rock types in a complicated terrain (as in our case). Given the multitude of spectral bands captured by sensors, PCA steps in as a dimensionality reduction technique, identifying and extracting the most significant patterns of variance within the dataset. The initial principal components generated through this process are particularly valuable because they encapsulate the largest portions of the data’s variability. In the context of lithological mapping, these first few principal components often condense crucial spectral information related to the distinct mineral compositions of various rock units. By creating color composites from these key components, subtle but diagnostic spectral differences between lithologies can be visually enhanced, leading to more accurate and robust interpretations of rock units and geological structures. The Minimum Noise Fraction (MNF) transform offers another sophisticated approach to handling the complexities of remote sensing data for applications like lithological mapping. Unlike PCA, which prioritizes variance, MNF focuses on separating the signal from the noise within the data. This is achieved through a two-step process involving two PCA rotations. Initially, MNF analyzes the noise characteristics of the dataset, decorrelating and rescaling the noise to create noise-whitened data. Subsequently, a standard PCA is performed on this noise-reduced data. The result is a set of transformed components ordered by decreasing signal-to-noise ratio, meaning the first MNF bands contain the most significant information with the least amount of noise. For lithological discrimination, these initial, high-quality MNF bands prove particularly valuable as they emphasize the subtle spectral signatures related to different rock types while effectively suppressing noise that could obscure these distinctions. MNF, akin to PCA, was used to assess the image data’s inherent dimensionality, filter out noise, and lower computational demands^54^. It condenses key components, ranking them from the most to least significant^55,56^. Independent Component Analysis (ICA) presents a distinct perspective on analyzing remote sensing data for lithological mapping by employing the principle of blind source separation. Unlike PCA, which seeks uncorrelated components with maximum variance, and MNF, which aims to maximize the signal-to-noise ratio, ICA strives to decompose the mixed signals within the remote sensing data into a set of components that are statistically as independent as possible. This is particularly useful in scenarios where the spectral signatures of different lithologies might be mixed or overlapping across various bands. By identifying these independent components, ICA can potentially isolate the unique spectral contributions of individual rock types, even without prior knowledge of the mixing process or the source spectral characteristics. This ability to disentangle mixed signals makes ICA a powerful tool for enhancing lithological discrimination, as it can reveal subtle spectral variations that might be obscured in the original data or by methods focusing solely on variance or noise reduction. Band math is the application of mathematical operations on chosen bands to highlight particular characteristics. A popular method that highlights potential alteration zones is band ratios, which divide one band’s digital number values by another. This generates a grayscale image that emphasizes relative band intensities, serving as a key indicator for certain types of alteration^57^. It is worth mentioning that several softwares have been utilized to accomplish this study (e.g., Geosoft Oasis Montaj 2015 v. 8.3.3, surfer 16.2, ENVI v. 5.6.2. and ARCGIS 10.8), full illustration of the methodologies is summarized in Fig. 3.

## Results

### Aeromagnetic results

Initially, to achieve decent and logical interpretations utilizing magnetic data, the effect of noise was reduced from the reduced to magnetic pole by applying upward continuation with level 200 m, Fig. 2b. Examination of the resulting map reveals a high magnetic anomalies with amplitude 42537.8 nT that oriented in NW, NE and E-W directions. The high magnetic sources are mostly linked to granite rocks, while the low magnetic anomalies with an amplitude of 42035.8 nT, occupy mainly the west part of the area investigated related to sedimentary rocks.

**Fig. 4:**
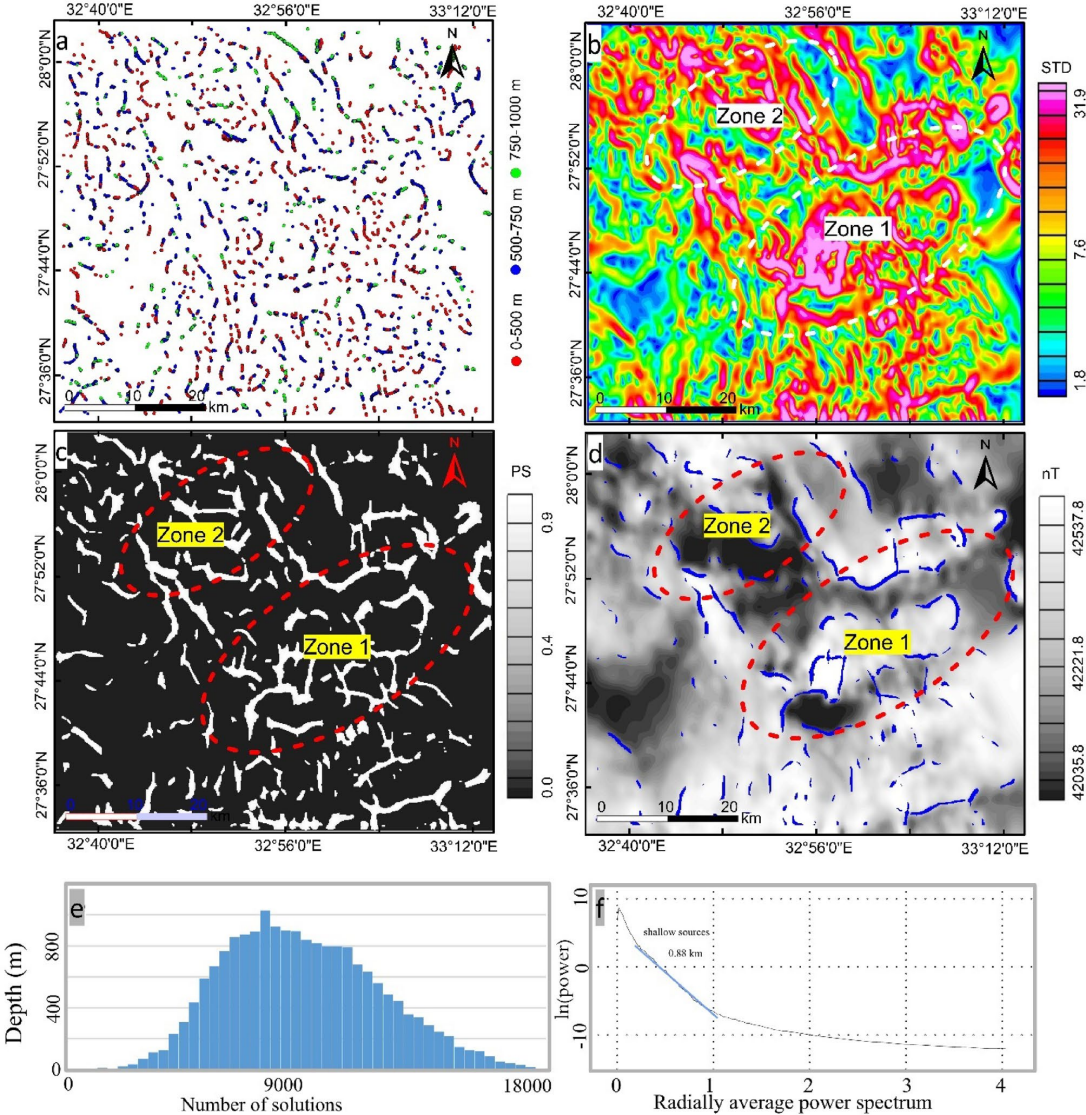
Structural complexity maps (**a**) Euler deconvolution depth solutions of a contact model (**b**) Standard deviation (STD) map, (**c**) phase symmetry map, (**d**) Vectorization overlain on grayscale RTP map, (**e**) histogram of euler solutions, (**f**) Power spectrum of RTP data Created by Geosoft Oasis Montaj 2015 v. 8.3.3 software; https://www.seequent.com/help-support/oasis-montaj/*).*

The analytic signal map (Fig. 2c) shows high magnetic zones occupied the central part of the study area and oriented in NW-SE trend, defining the vital direction that controls mineral occurrence in the investigated area. These high magnetic zones represent areas of potassic alteration related to porphyry intrusions. The first vertical derivative map (Fig. 2d) gives more details about the appearance of structures in the area. The map exhibits NW-SE, NE-SW, and N-S directions. The depths of these solutions in terms of contacts are shown in the Euler deconvolution depth map (Fig. 4a). Based on this map and the constructed histogram (Fig. 4e) the depths fall between 0 and 800 m exhibiting shallow sources, coinciding with the results of power spectrum^58^ Fig. (4f). The depth solutions in some areas appeared as circular shapes give a good impression about the occurrence of circular porphyry features in the area specially in the southeast part.

Unveiling the structural complexity of the area in terms of texture, phase and lineations analysis is performed by CET grid which includes the Standard deviation (Fig. 4b) of the RTP data. The STD map heights the local variations associated with magnetic discontinuity and represented by a high value of standard deviation (pink color). Two zones are detected on the STD map visualizing the regions of local complex structures, occupied Gabal monqul, south Gabal monqul, Umm Balad, and northwest Dara areas. Following that the lateral continuous line- likes zones of complex texture are isolated by applying phase symmetry on the STD map (Fig. 4b).

**Fig. 5:**
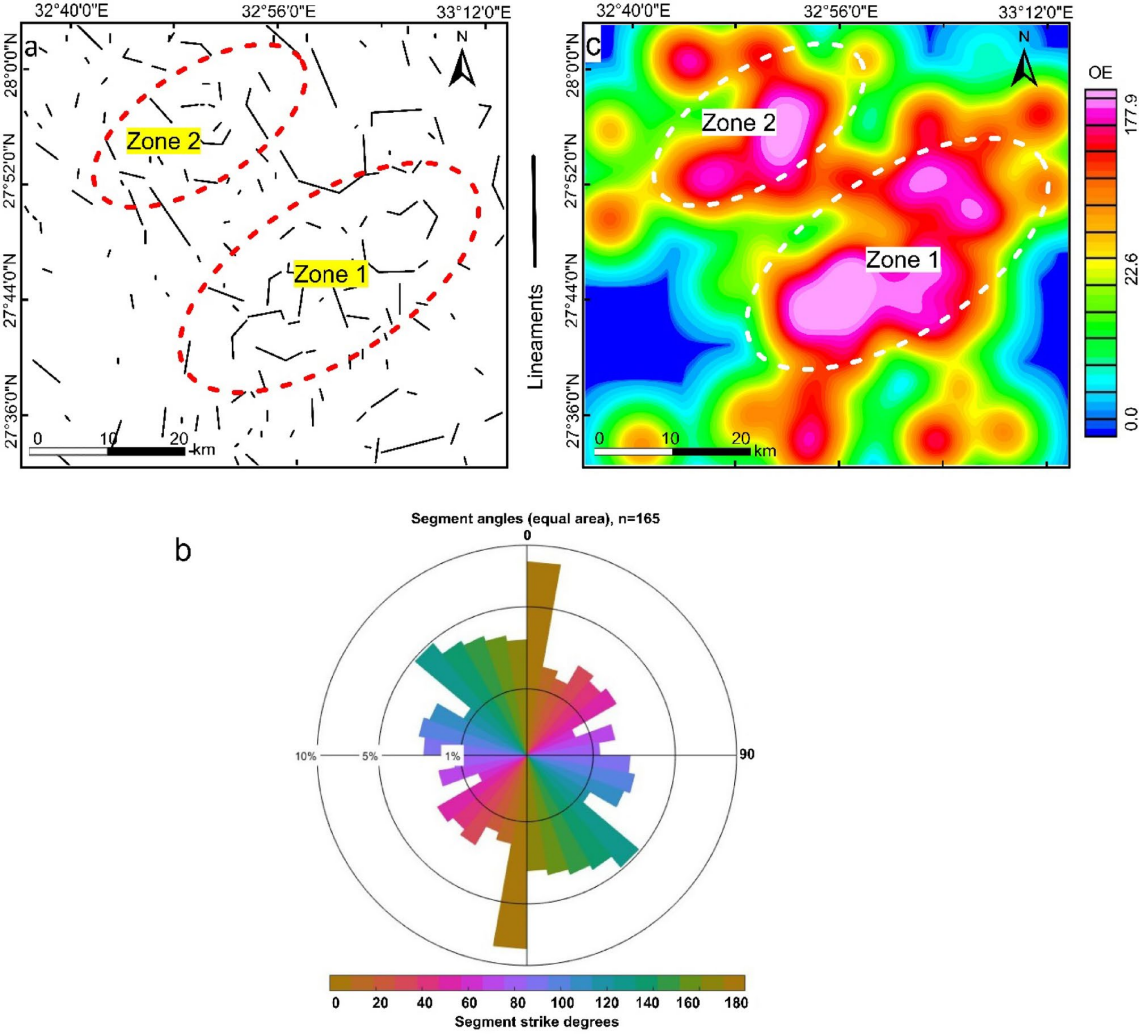
Structural complexity maps (**a**) vectorization lineaments map, (**b**) Rose diagram showing the number of lineaments (*n* = 165) with strike degree generated by using FracPaQ MATLAB toolbox, (**c**) orientation entropy heat map. Created by Geosoft Oasis Montaj 2015 v. 8.3.3 software; https://www.seequent.com/help-support/oasis-montaj/*).*

The obtained phase symmetry map (Fig. 4c) picks out regions of magnetic discontinuity features such as contacts, faults, dykes and lithological boundaries. These features are mostly seen to situate the two mapped zones (Fig. 4d). Then a set of filters containing amplitude thresholding, skeletonization and skeleton to vectors are applied successively on the Phase Symmetry map. The result of these procedures is a vectorization map (Fig. 5a), exhibiting automated linear structures of the targeted area. A momentary look at the map we can observe that most of the produced lineaments are oriented in NW, and NE directions, controlling the occurrences of ore deposits in Egypt. Statistical analysis in terms of rose diagram, indicating a number of lineaments and strike degree of each one has been done by using FraqbaQ2D^59^ as shown in figure (5b). Furthermore, the previously spotted two zones show much more complex structures. The variation in the orientation of the detected features could be translated into a complex heat map in terms of orientation entropy (Fig. 5c), manifesting the two optimistic zones of mineral occurrences.

**Fig. 6:**
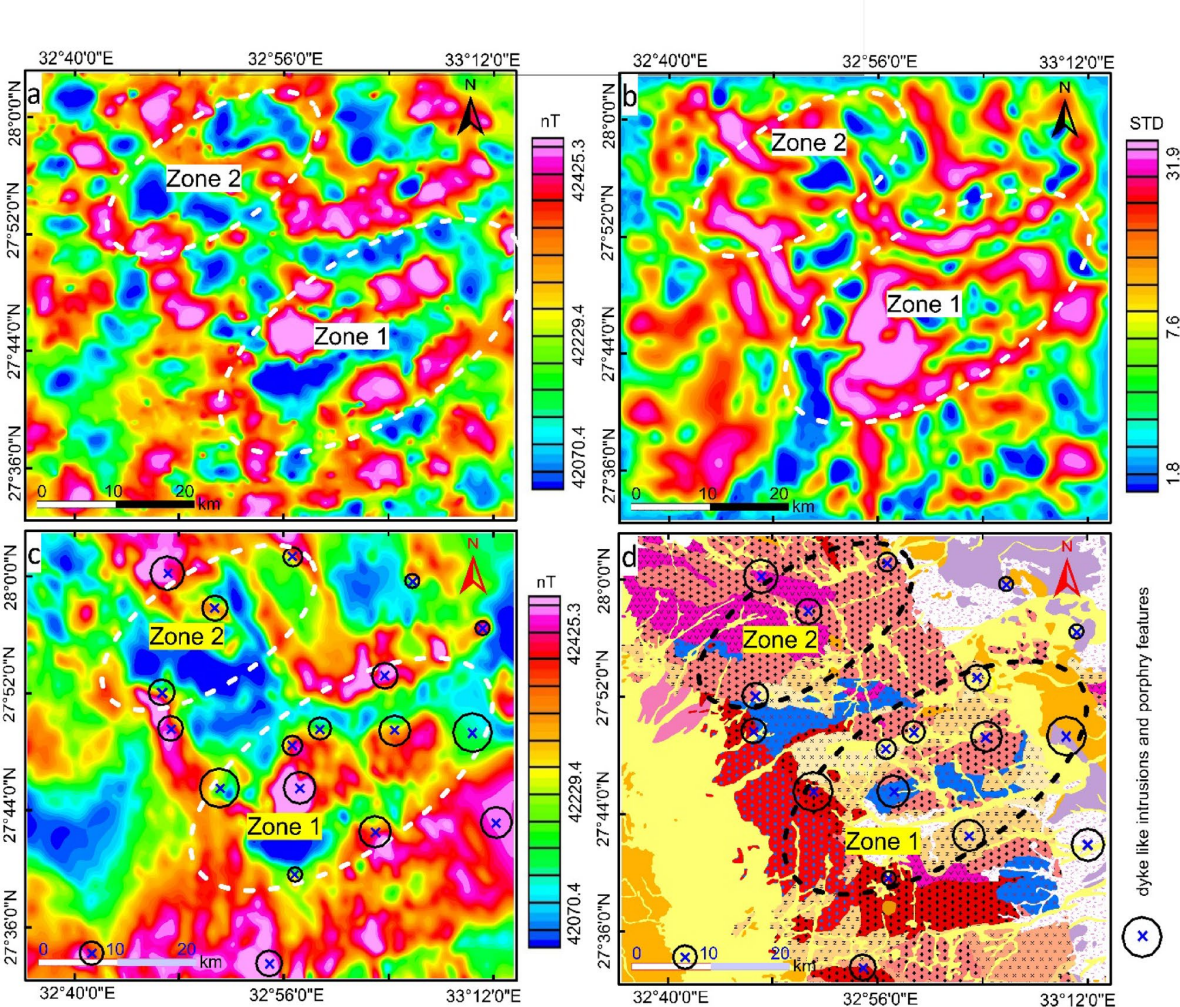
Porphyry detection maps, (**a**) Circular feature transform, (**b**) Amplitude contrast transform, (**c**) detected circular porphyry feature and their centers overlain RTP map, (**d**) Detected circular porphyry feature and their centers overlain geological map of Dara - Monqul area, Northern Egyptian Eastern Desert, modified after^45^. Created by Geosoft Oasis Montaj 2015 v. 8.3.3 software; https://www.seequent.com/help-support/oasis-montaj/*).*

In view of previous studies, areas rich in porphyry-type mineralization were recognized, which are located south of Gabal Monqul. By applying CET porphyry approach to aeromagnetic measurements, we have been able to identify and monitor the locations of the dyke-like intrusions and porphyry styles, as well as the new locations that are likely to be promising. What has been already implemented to define these tracts is that the Circular feature transform filter was applied to RTP data to show up places of the circular feature and their centers (Fig. 6a), then the boundary of these circular features are determined by applying amplitude contrast transform (Fig. 6b). Lastly, visualizing the results is performed by plotting the detected circular and their centers (Fig. 6c). To exactly underline the locations of the detected dykes and porphyry features, the results were also overlain on the geological map of the researched area (Fig. 6d). Based on this map we can conclude that most of the exposed dyke like intrusion and porphyry features, associated with hydrothermal alteration related (Cu-Au) mineralization is situated in the previously mapped two favorable zones.

### Remote sensing results

**Fig. 7:**
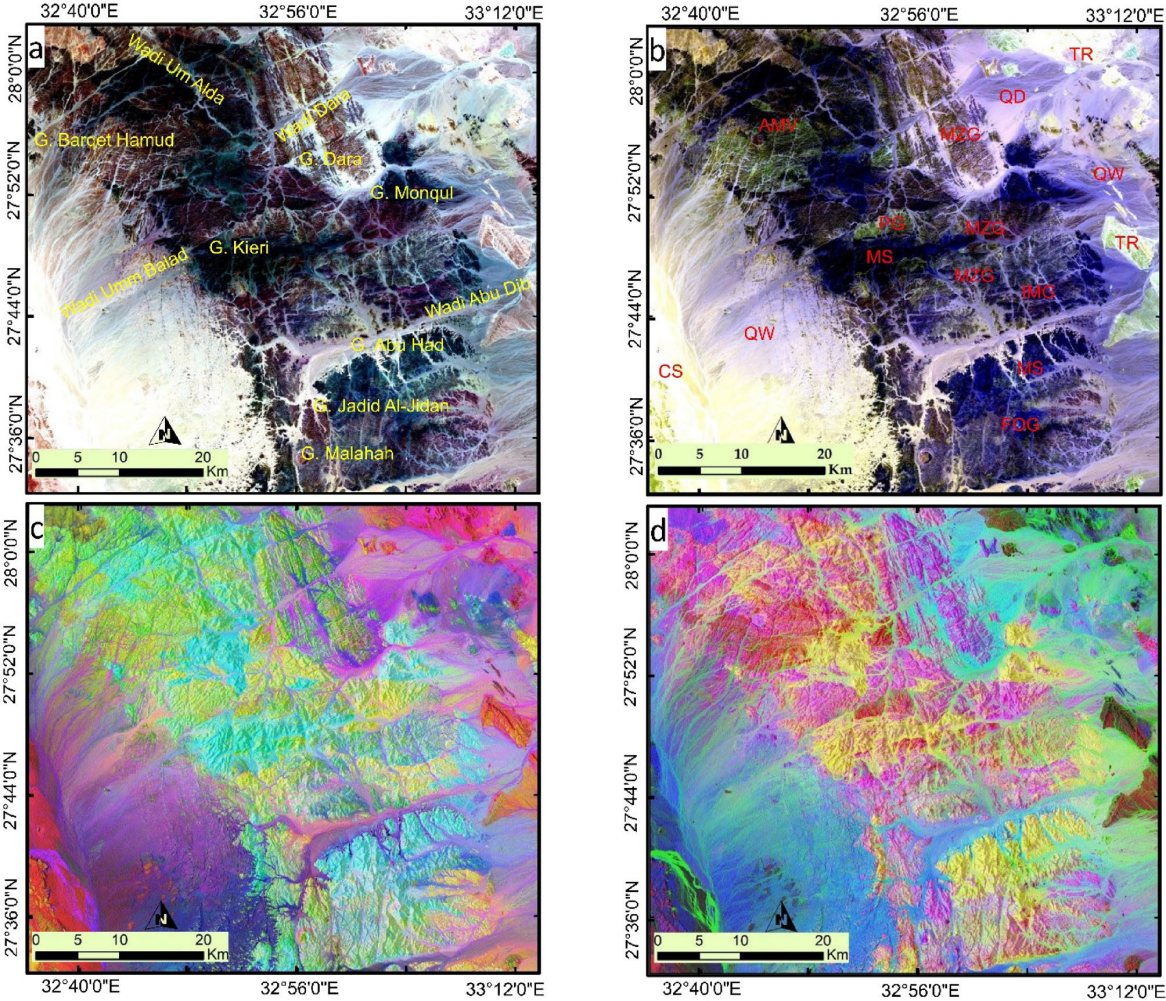
Illustrates the general lithological discrimination of the studied rock units through prepared FCC using Sentinel-2 data of (**a**) 12-6-2 in RGB and (**b**) 12-11-2 in RGB highlighting the contrast between granitic rocks and metavolcanics besides clear depiction of wadi deposits and evaporites. (**c**) PC3-PC1-PC2 in RGB and (**d**) PC1-PC2-PC5 in RGB highlighting all the lithological units within the studied terrain. Created by ArcGIS Desktop 10.8. https://www.esri.com/en-us/arcgis/products/arcgis-desktop/overview, and ENVI v. 5.6.2. software; (https://www.l3harrisgeospatial.com/Software-Technology/ENVI*).*

The image processing results revealed several findings that boosted the geological understanding of the study area. Figure 7 illustrates the general lithological discrimination of the studied rock units using Sentinel-2 data. Figure 7a highlights that the Precambrian basement rocks form an elongated sector between G. Malahah (south) and W. Um Alda (north), predominantly surrounded by Phanerozoic rock and wadi deposits to the east and west using the band combinations of bands 12-6-2 (RGB). Both Fig. 7a and b (bands12-11-2 in RGB) underscore the lithological complexity of the terrain, clearly distinguishing the G. Dara granitic rocks, represented by a brown color and characterized by their elliptical, northwest-trending shape. Just south of G. Dara, the G. Monqul area is distinguished by a dark pinkish color, indicative of monzogranites, as shown in Fig. 7a.

The distribution of granitic rocks is better depicted in Fig. 7c through band combinations of PC3-PC1-PC2 in RGB, where they are represented in yellow to yellowish-green hues. Meanwhile, metavolcanics and metasediments, which constitute a significant portion of the study area, are easily distinguished by cyan to cyanish-green tones. In Fig. 7d (PC1-PC2-PC5 in RGB), these rocks are highlighted in yellow, while the granitic rocks appear in varying shades of pink, reflecting their differing compositions. For instance, the tones of G. Dara and G. Monqul differ from each other in the eastern part of the study area. Acidic metavolcanics around G. Barquet Hamud are displayed in a reddish-pink color and are clearly differentiated from other granitic rocks. However, the pinkish color is still represented in that component for these rocks reflecting their acidic compositions.

**Fig. 8:**
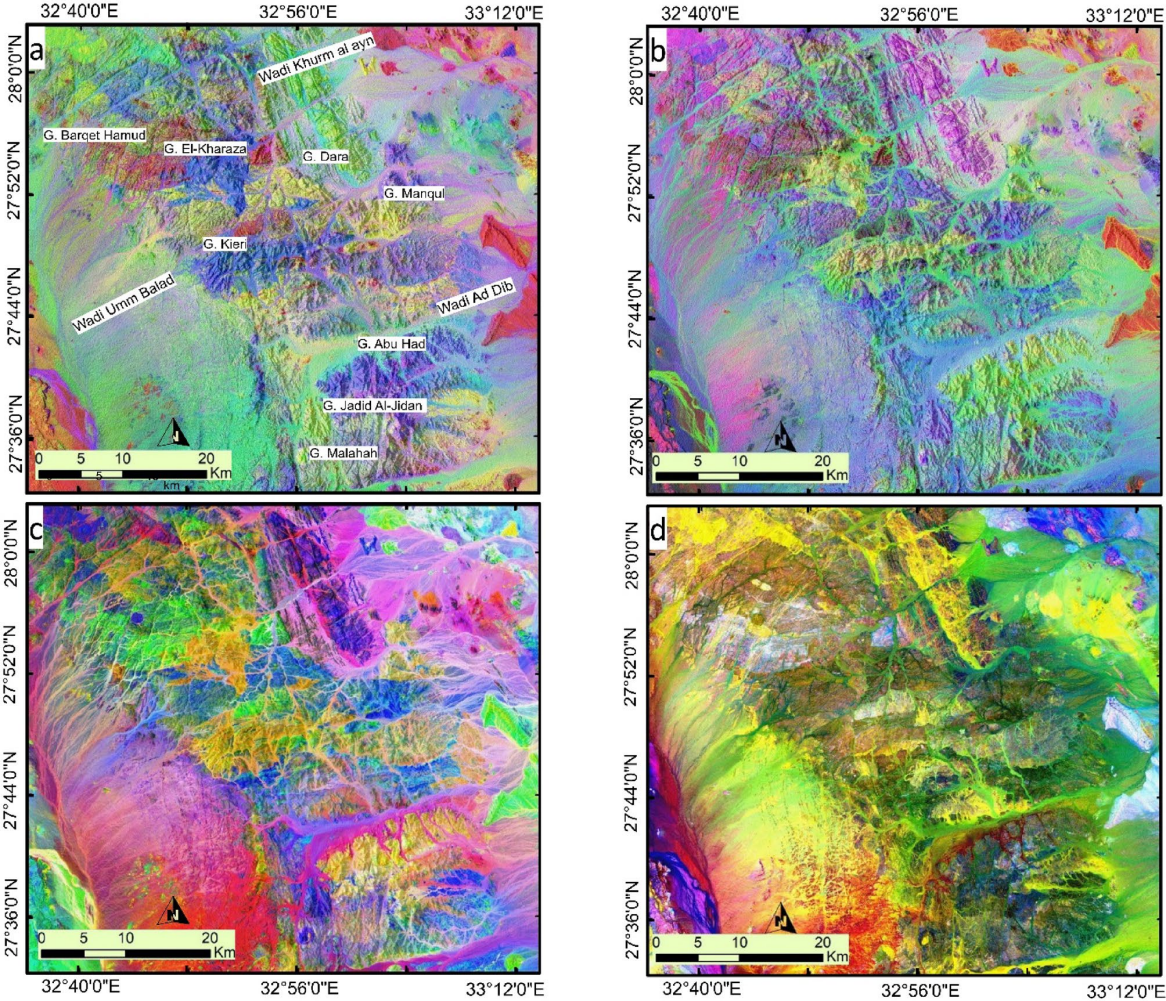
Detailed Lithological discrimination using Sentinel 2 Combinations of (**a**) PC3-PC5-PC2 in RGB and (**b**) PC6-PC2-PC5 in RGB highlighting the lithological complexity of the study area and the lithological heterogeneity even within the same rock unit. Lithological discrimination using ASTER (**c**) MNF4-MNF2-MNF3 in RGB, and (**d**) IC2-MNF1-IC1 in RGB, highlighting the structural complexity of the study area especially related to the granitic rocks. Created by ArcGIS Desktop 10.8. https://www.esri.com/en-us/arcgis/products/arcgis-desktop/overview, and ENVI v. 5.6.2. software; (https://www.l3harrisgeospatial.com/Software-Technology/ENVI*).*

A more detailed differentiation of the main lithological groups (granites, metavolcanics, and metasediments) is presented in Fig. 8 using PCA, ICA, and MNF. For example, using Sentinel-2 band combinations, Fig. 8a (PC3–PC5–PC2 in RGB) and Fig. 8b (PC6–PC2–PC5 in RGB) clearly distinguish the G. Dara granite (alkali-feldspar granite) from the G. Monqul (Dokhan volcanics). For instance, Sentinel-2 band combinations used in Fig. 8a (PC3–PC5–PC2 in RGB) and Fig. 8b (PC6–PC2–PC5 in RGB) effectively differentiate the G. Dara granite (alkali-feldspar granite) from G. Monqul (Dokhan volcanics; calc-alkaline andesitic to rhyolitic rocks), G. Jadid Al Jidan (older granite; calc-alkaline, foliated quartz dioritic to granodioritic rocks), and G. Malahah (Dokhan volcanic rocks). The intrusive metagabbro is identified by a dark blue color. The acidic metavolcanics in the western part of the study area are represented by a deep red tint. Figure 8c is considered one of the best combinations and can be regarded as an updated lithological map of the studied terrain, highlighting the boundaries between different rock units. For instance, the granitic rocks, in all their variations, are shown in shades of blue, with varying intensities. The intrusive gabbroic rocks are depicted in yellow, while the metavolcanics and metasediments are characterized by green hues. Using ASTER data, the main acidic metavolcanic block appears as a bright green area in the MNF4–MNF2–MNF3 RGB combination (Fig. 8c) and is represented by the brightest pixels among all rock units in Fig. 8d, as shown using the IC2–MNF1–IC1 RGB combination.

**Fig. 9:**
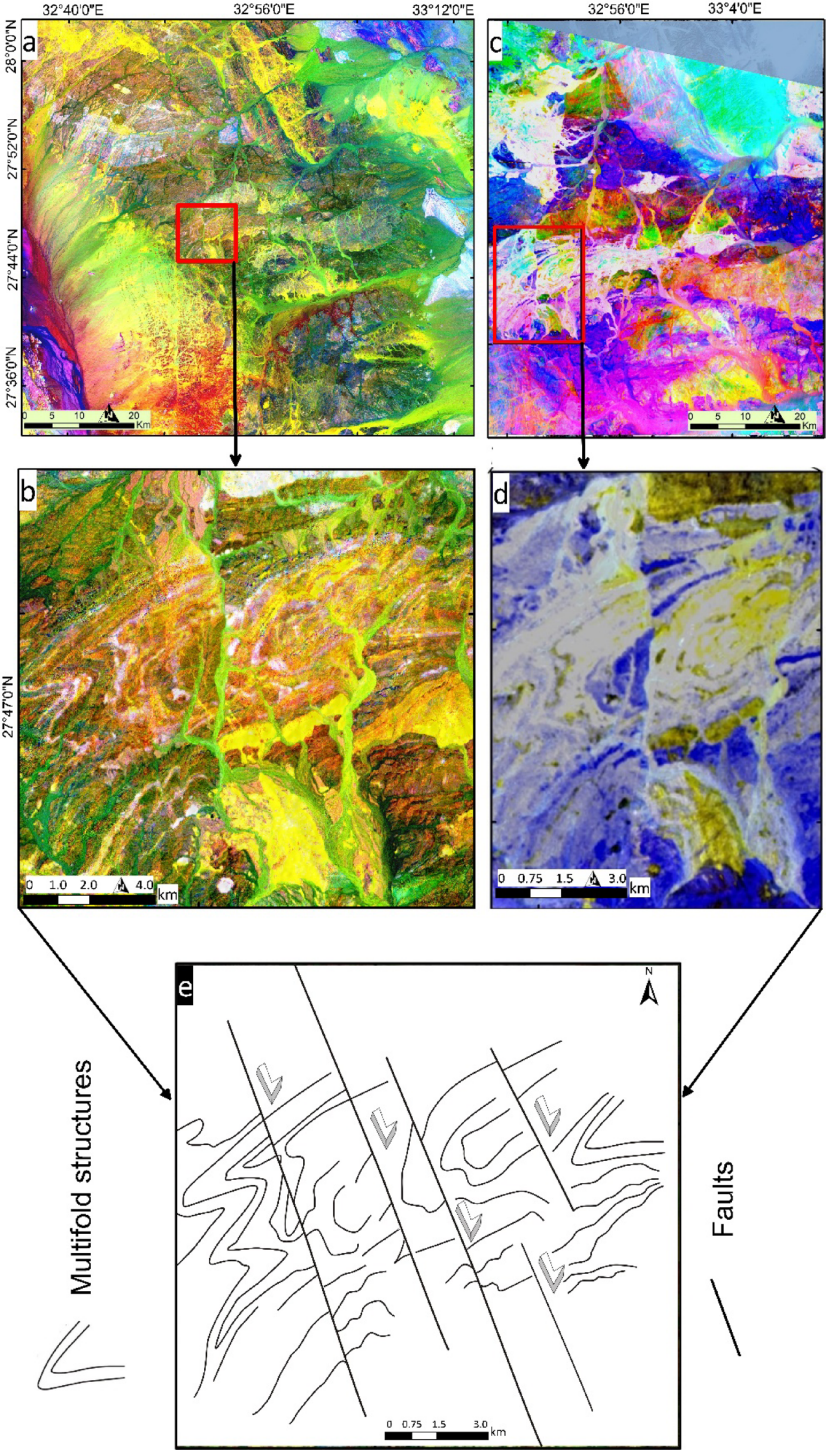
(**a**) IC2-MNF1-IC1 in RGB, (**b**) A close-up view of the central part of the study area, showcasing the lithological and structural complexity and highlighting multifolding by the bright pinkish white pixels through IC2-MNF1-IC1 in RGB respectively, A close-up view using PRISMA hyperspectral data, (**c**) MNF8- MNF6-MNF9, (**d**) MNF6-MNF6-MNF9 in RGB respectively, showcasing the lithological and structural complexity within the study area, and (**e**) Detailed sketched multifold structures. (Created by ArcGIS Desktop 10.8. https://www.esri.com/en-us/arcgis/products/arcgis-desktop/overview, and ENVI v. 5.6.2. software; (https://www.l3harrisgeospatial.com/Software-Technology/ENVI*).*

The key structural features of the study area are shown Fig. 9a. A close-up view of Fig. 9a (IC2-MNF1-IC1 in RGB) is presented in Fig. 9b, focusing on the structural complexity in different parts of the study area (highlighted by red box). Figure 9b emphasizes the linear structures along the wadis and the NE-trending features, revealing both lithological and structural complexity. Additionally, the image highlights areas of intense folding, marked by bright pinkish-white pixels, using the IC2-MNF1-IC1 bands in RGB.

PRISMA hyperspectral data provided enhanced discrimination of the most important sections of the study area, as demonstrated by previous methodologies. Figure 9c shows the intrusive gabbro in a bright color, while the metavolcanics are blue. In addition to lithological differentiation, Fig. 9d (MNF6-MNF6-MNF9 in RGB) includes a close-up view of the western part, which not only highlights the multifold structures but also reveals the lateral displacement of these folded rocks. Detailed sketched multifold structures are presented in Fig. 9e. Structurally, the area has undergone both ductile and brittle deformation. The lithologies are folded, with nearly vertical axial planes striking NE-SW, reflecting NW-SE-directed shortening at deep crustal levels. Subsequently, the region was cut by NW-SE strike-slip faults at shallow crustal level with dextral displacement, crosscutting the pre-existing folds.

All the previous results demonstrate the lithological and structural complexity of the studied area, which, in most cases, is responsible for the hydrothermal alteration activities observed. These hydrothermally altered zones are of particular importance as they are considered potential sites for mineralization, especially when coinciding with significant structural features.

**Fig. 10:**
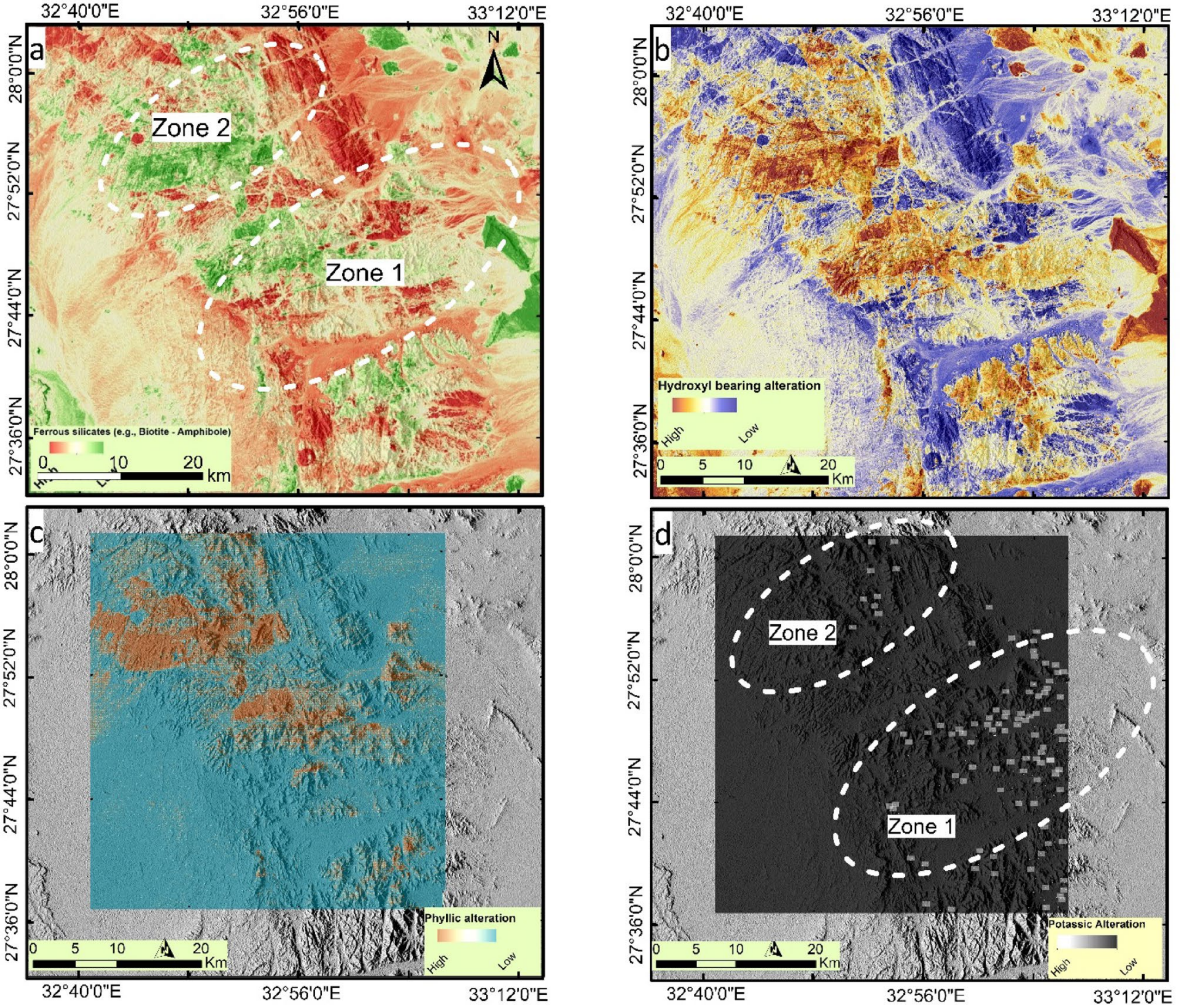
Hydrothermal alteration mapping showing, (**a**) Ferrous silicates using Sentinel 2 (b12/b11), (**b**) OH bearing minerals using Sentinel 2 (b11/b12), (**c**) Phyllic type using ASTER (b5 + b7/b6) overlaid on hill shade map, (**d**) Potassic type using ASTER ((b10*b12)/(b11*b11)) overlaid on hill shade map of the study area. Created by ArcGIS Desktop 10.8. https://www.esri.com/en-us/arcgis/products/arcgis-desktop/overview, and ENVI v. 5.6.2. software; (https://www.l3harrisgeospatial.com/Software-Technology/ENVI*).*

Therefore, Sentinel-2 data and ASTER combinations were applied, following widely recognized band math, to monitor hydrothermal activity. The following band equations are widely employed in remote sensing for geological mapping and mineral exploration. The ratio of Band 12 to Band 11 (b12/b11) is commonly used to detect ferrous silicates, as demonstrated by^20,60^. In contrast, the inverse ratio (b11/b12) is utilized to map hydroxyl-bearing minerals (e.g. kaolinite, illite, epidote and chlorite), as noted by^20,61^. For the identification of phyllic alteration, the ratio (b5 + b7)/b6 is applied, following the methodologies outlined by^20,61^. Finally, potassic alteration is delineated using the equation ((b10 × b12)/(b11 × b11)). Figure 10 presents these results, showing the distribution of ferrous silicates (Fig. 10a) and OH-bearing minerals (Fig. 10b) using Sentinel-2 data. Figure 10a shows the extensive distribution of ferrous silicates, which primarily represent biotite and amphibole. These minerals are essential components of granitic rocks and are sometimes associated with porphyry alteration zones. Figure 10b shows the distribution of OH-bearing minerals (e.g. kaolinite, illite, epidote and chlorite) which are considered associated with various types of alterations. Upon comparing our highly altered zones with previous research over the study area, most of the known potential zones spatially coincided with high OH-bearing content besides highlighting new alteration zones that may be considered potential future explorations for Cu-Au.

A more detailed analysis was conducted using ASTER data to highlight the primary alteration types commonly associated with porphyry mineralization deposits. Previous results indicate a strong correlation between porphyry mineralization and phyllic and potassic alteration types. These zones reflect different temperatures and fluid conditions. Phyllic alteration is characterized by the assemblage of sericite, quartz, and pyrite, typically forming along fault zones and shear planes within metavolcanic rocks and at the margins of granitic intrusions. In contrast, potassic alteration, dominated by secondary K-feldspar and biotite, is commonly observed proximally to the granitic bodies. These alteration zones are spatially associated with structurally controlled mineralization, particularly along major faults and lithological contacts^40^. The distribution of the phyllic type (Fig. 10c) aligns with the zones identified by geophysical findings, emphasizing their higher potential for mineralization. In contrast, the distribution of potassic alteration is mostly confined to the eastern part of the study area, with a few scattered pixels in the northern zone (Fig. 10d).

## Discussion and validation

**Fig. 11:**
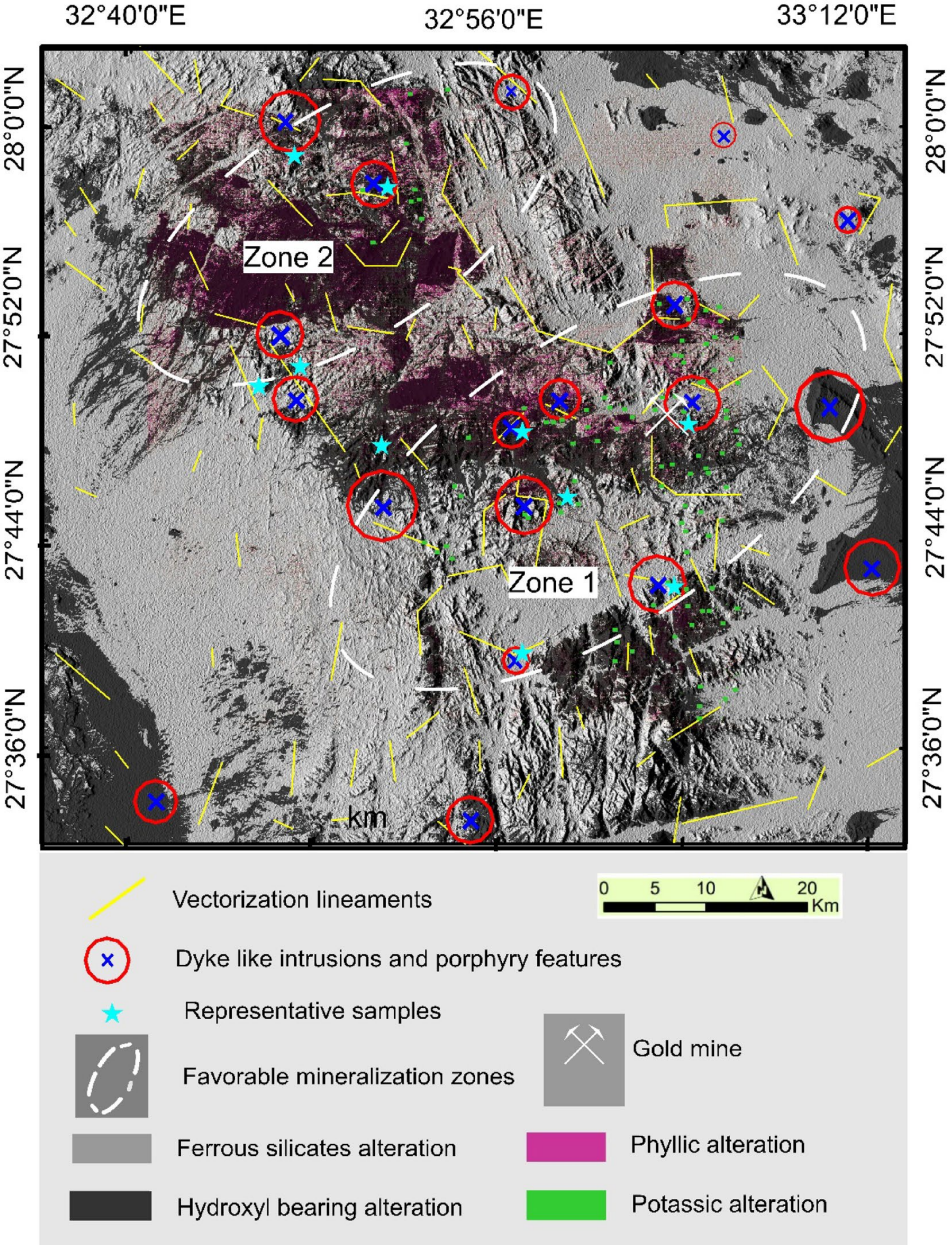
Composite potential mineralization (CPM) map of the study area. Created by ArcGIS Desktop 10.8. https://www.esri.com/en-us/arcgis/products/arcgis-desktop/overview, and Geosoft Oasis Montaj 2015 v. 8.3.3 software; https://www.seequent.com/help-support/oasis-montaj/*).*

Once a considerable number of criteria are gathered at one location, it implies a high possibility of mineral deposits, particularly those connected with the porphyry type. Consequently, a composite potential mineralization (CPM) map (Fig. 11) was constructed based on the interpretation of Aeromagnetic and Remote sensing data. A close inspection of the map, exhibiting a number of lineaments about 165 made the area highly deformed and sheared. These lineament trends follow NW, NE, NNE, and N-S representing pathways for ore fluids. The NW-SE, known as the Najd fault system, plays a vital role in controlling mineralization occurring in the area^16,62^. Two promising zones (1 & 2) of mineralization are detected in the investigated area. Zone No. 1 covers around 454km^2^ and is located in the eastern part occupied by Gebel Monqul, and the surroundings were very rich in the possibility of the presence of porphyry-type minerals due to the availability of various hydrothermal variations, especially potassium and phyllic types. Also, the structure complexity, dyke like intrusion and circular porphyry features vary in size are more abundant in this part, as well as the existence of one of the ancient gold mines. Zone No. 2 with an area of approximately 283km^2^ is situated in the north part of the area around Umm Balad and Northwest Gebel Dara is characterized by phyllic alteration with structure complexity and porphyry features.

**Fig. 12:**
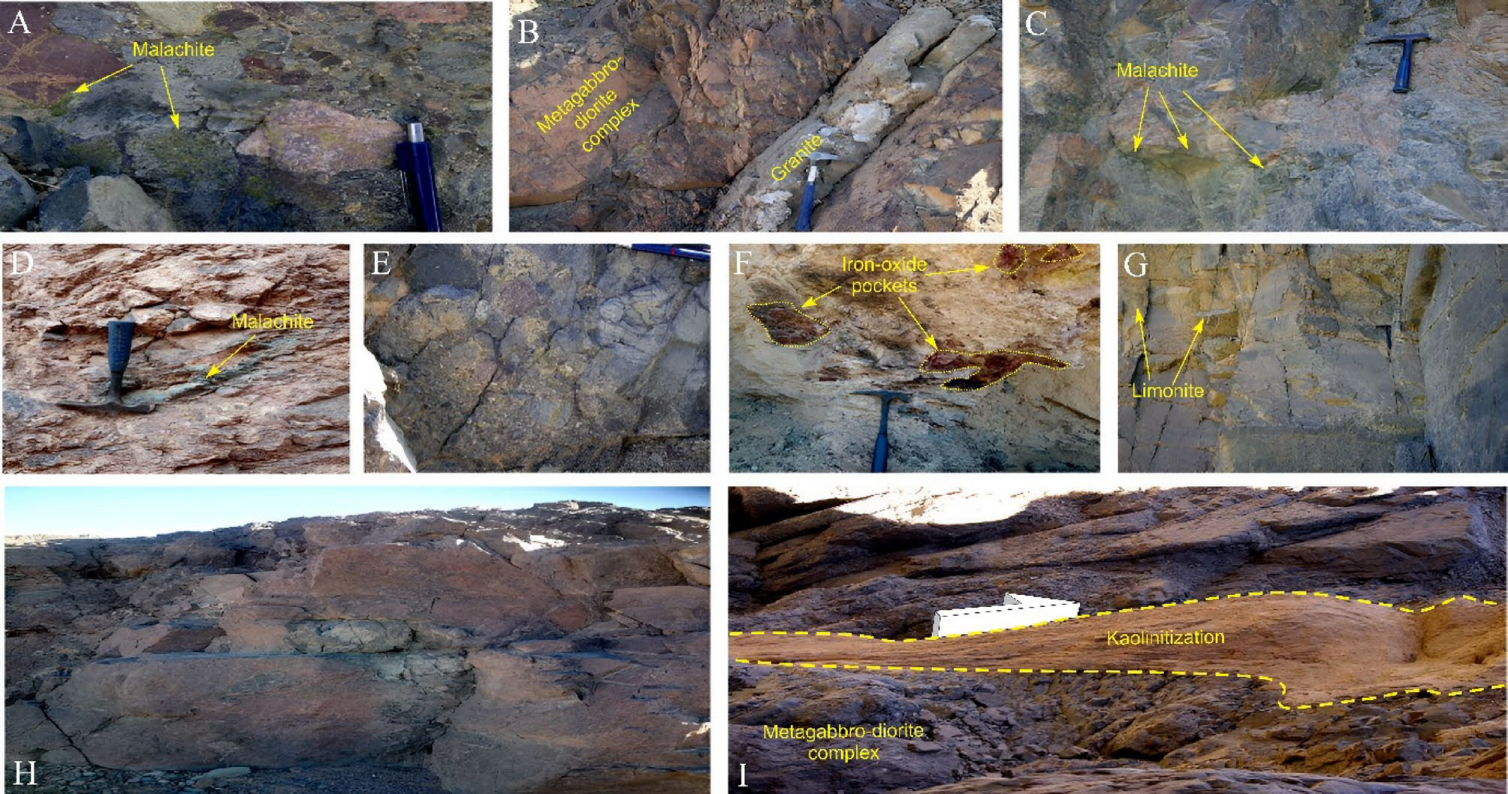
Field observations from the study area illustrating key features associated with potential mineralization: (**A**) Island arc-related pyroclastic units, including laminated ash, lapilli, crystal-rich metatuffs, ignimbrites, and agglomerates; (**B**) Sharp intrusive contact where a granodioritic intrusion cross-cuts the pre-existing gabbro-diorite complex; (**C**) Sulphide-iron oxide and malachite-enriched gabbroic rocks; (**D**) Felsic dyke intruding tonalite-granodiorite rocks, locally preserving thin, malachite-stained fractures; (**E**) Alteration zones developed within highly fractured metavolcanic rocks; (**F**) Sulphide-iron oxide mineralization hosted in talc schist; (**G**) Limonite-bearing altered zone; (**H**) Intensely fractured metagabbro-diorite complex; and (**I**) Kaolinized, Z-shaped structural enclave displaced along a dextral shear zone affecting the metagabbro-diorite sequence.”.

A field examination was carried out to verify lithology, evaluate geological features, investigate the linkages between exposed rock units and detect probable mineralized zones (1 & 2). Potential mineral occurrences in the research area are hosted within the extensively distributed metagabbro-diorite rocks, which contain malachite and sulfides, as presented in Fig. 12. Alteration minerals are visible along zones of weakness and fractures, where the rocks have been primarily sheared and fractured. These rocks exhibit blocky weathering, minimal deformation, and weak banding. The mineral composition of the metagabbro-diorites varies from diorite to gabbro and, in some areas, quartz diorite, depending on the proportions of feldspar, mafic minerals, and quartz present. Mineral-bearing alteration zones have developed nearby, with concentrations of sulfides, malachite, and iron oxide, as well as potential gold-bearing sulfides and malachite.

Notable alteration processes, such as hematization, kaolinization, and silicification, are evident at the contact between the quartz veins and the surrounding rocks. These alteration zones range in width from 8 to 15 m and are often characterized by numerous quartz swarms, veinlets, and lenses. Their composition predominantly includes pyrite cubes, calcite, sericite, kaolin, smoky quartz, and scattered goethite.

For detailed mineralogical analysis, Scanning Electron Microscopy (SEM) investigations were carried out using a Zeiss SmartEDX system. Ten representative samples (S1–S10) were systematically collected from key alteration zones and quartz veins identified during fieldwork. Sampling locations were selected based on visible alteration features such as hematization, kaolinization, silicification, and the presence of sulfide mineralization. Each collected sample was carefully cleaned, air-dried, and mounted on aluminum stubs using carbon tape, then coated with a thin layer of gold to enhance surface conductivity prior to SEM examination. SEM analyses were performed under an accelerating voltage of 20 kV, at a magnification of approximately 500×, and a takeoff angle of 35°. The Zeiss SmartEDX system was equipped with an Energy-Dispersive X-ray Spectroscopy (EDS) detector, with measurements recorded using a live time of 202.8 s, amplifier time of 3.84 µs, and a resolution of 128.6 eV.

**Fig. 13:**
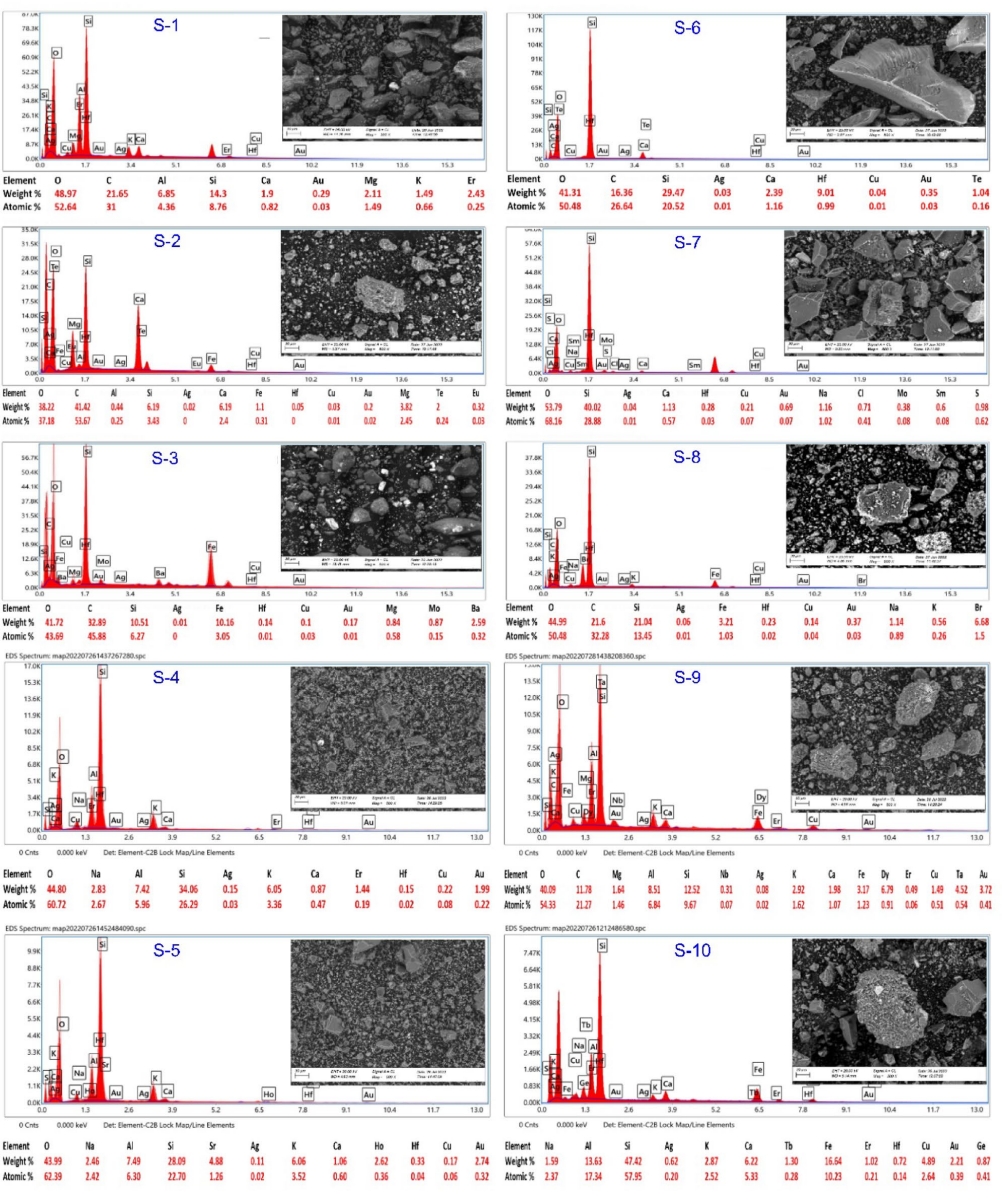
SEM images, corresponding sum spectrum, and elemental composition of representative rock samples.

The SEM investigation focused on identifying the micro textures and mineral phases associated with the alteration processes and mineralization. Elemental mapping and sum spectra were used to quantify the elemental concentrations, revealing significant Ag, Fe, Si, K, and Al contents, with trace amounts of Au and Cu in some samples. The results, including high-resolution SEM images, elemental overlays, sum spectra, and quantified elemental compositions, are presented in Fig. 13. These laboratory findings were cross-referenced with field observations to validate the nature and distribution of alteration and mineralization zones.

The analysis of representative rock samples (S1–S10) indicates significant Cu and Au mineralization, within the two promising Zones. As illustrated in Fig. 13, some samples exhibit high weight% values for both copper and gold. Zone 1 (S3, S4, S7, S9, and S10) demonstrate markedly high Cu and Au contents compared to Zone 2 (S1, S2, S5, S6, and S8). For instance, sample S10 from Zone 1 recorded the highest copper content at 4.89 wt% Cu and 2.21 wt% Au, followed by sample S9 with 1.49 wt% Cu and 3.72 wt% Au. In contrast, samples from Zone 2 yielded lower values, such as S1, with no detectable copper and only 0.29 wt% Au, and S2, with 0.03 wt% Cu and 0.20 wt% Au. These geochemical results support the greater mineralization potential of Zone 1 and validate field-based observations and petrographic evidence.

## Conclusion

- Multi sources data including aeromagnetic, ASTER, SENTINEL, PRISMA, Field observation and SEM have been utilized to assess the existence of mineralization (porphyry Cu-Au) by identifying evidence and associated characteristics.
- Aeromagnetic data analysis in terms of AS, FVD and ED largely indicates the preferred direction controlling the mineralization occurrences, with estimating their depths. These structures are oriented in the direction of NW-SE, NE-SW, NNE-SSW and N-S, with depth ranges from 0 up to 1 km. The CET grid, and porphyry analysis positively defined the structure complexity, dyke like intrusions and circular to semicircular porphyry structures, defining highly altered rocks.
- FCC, PCA, ICA, MNF, and BR of Remote sensing data successfully contributed to lithological discrimination. The prospect area is primarily composed of monzogranite, dacite, and minor hornblende gabbro. The dacite, interpreted as part of the Dokhan Volcanics, is porphyritic and exhibits alteration to a chlorite-carbonate-sericite assemblage, indicative of greenschist-facies metamorphism or post-magmatic alteration. Various types of alteration that are more related to porphyry mineralization, involving phyllic, potassic, ferrous silicates and hydroxyl bearing are well mapped.
- Two zones (1&2) have been recognized with a significant probability of mineralization (Cu-Au) occurrence. Field observation, and SEM analysis of ten representative samples have been performed to verify these zones.
- To enhance exploratory efforts, geochemical analysis, geoelectric (e.g., Induced polarization) and Electromagnetic (EM) technologies can provide valuable insights into underlying mineralization. This study, together with previous studies and other methods recommended for future use, may open new horizons in mineralization in Egypt.

## Data Availability

The datasets used and/or analyzed during the current study are available from the corresponding author upon reasonable request.
